# Supplementary material for: Modulation of the mTOR pathway plays a central role in dendritic cell functions after Echinococcus granulosus antigen recognition
Source: Sci Rep. 2021 Aug 26;11:17238. doi: 10.1038/s41598-021-96435-z (PMC8390662; doi:10.1038/s41598-021-96435-z)
Supplement: Supplementary file 1 — Supplementary Figure 1. [file 41598_2021_96435_MOESM1_ESM.pdf]

Modulation of the mTOR pathway plays a central role in dendritic cell functions after Echinococcus granulosus antigen recognition.

Christian Rodriguez Rodriguez, María Celeste Nicolao, Maia Chop, Natalia Plá, Mora Massaro, Julia Loos, Andrea C. Cumino.

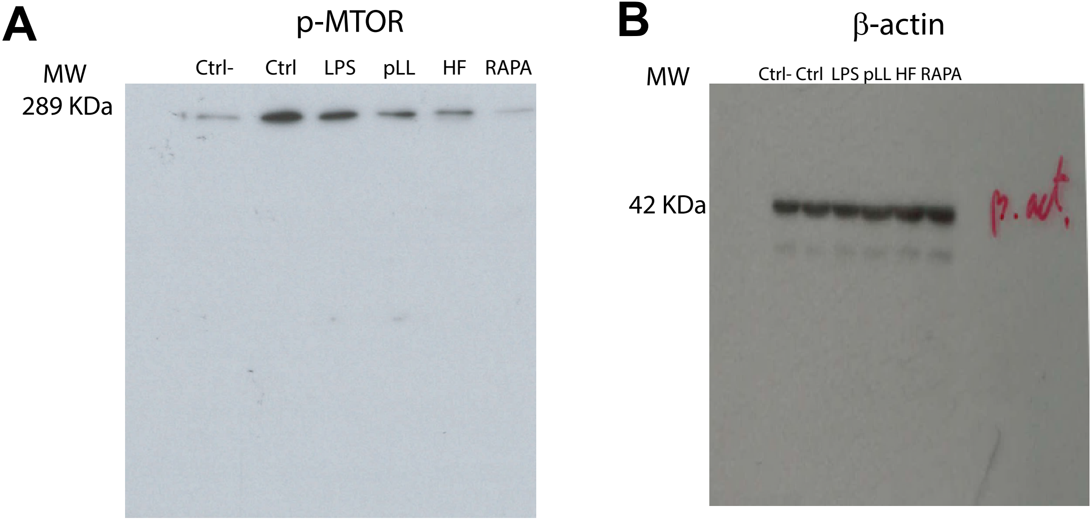

Supplementary Figure 1

A- Western blot analysis of extracts from BMDCs (1x106/ml), untreated, stimulated for 18h with 100 ng/ml LPS or Eg antigen-treated (20 μg pLL or 5 μg HF), using Phospho-mTOR (Ser2448) (D9C2). B- Immunoblot of β-actin is shown. It was used for equal loading control.
